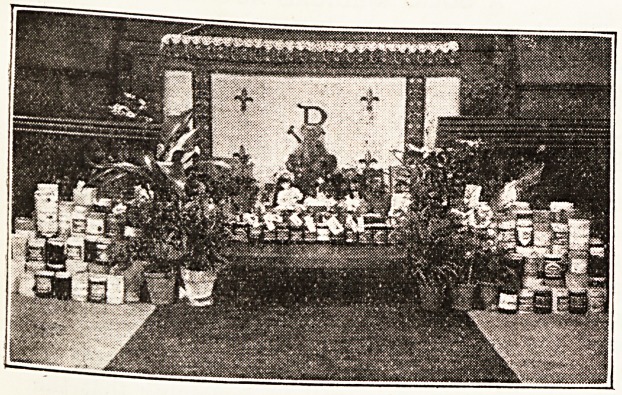# Hospital and Institutional News

**Published:** 1913-04-05

**Authors:** 


### HOSPITAL AND INSTITUTIONAL NEWS.

### INSURANCE PATIENTS IN REVOLT.

On behalf of the Welsh Insurance Commissioners, Dr. D. Llewelyn Williams has held an inquiry into the condition and administration of the Udal Torre Sanatorium, South Devon, from which, as we reported last week, eleven patients "revolted," alleging that the food was bad and other grievances. Dr. Llewelyn Williams states: "As far as I can see the treatment and methods adopted are on the right lines, and I think it would be a pity if this incident, which reflects so unfairly on the staff, should interfere in any way with the very successful work carried on there." On a surprise visit to the sanatorium Dr. Williams discussed matters with the remaining patients, one of whom, we learn, spontaneously proposed a vote of confidence in Dr. H. E. Watson, the medical superintendent, which was carried. On the day of the revolt the porridge was admittedly burnt, and the ham "a little underdone," but Dr. Williams was convinced that the complaints about the food were exaggerated. As to the washing of cups and plates, Dr. Williams says that it is the custom here, as at every well-conducted sanatorium, for each patient to wash his own, which are numbered. The men did not object to cleaning their rooms, but the scrubbing of the dining-room floor would be done by maids in future. Dr. Williams sums up the case by saying: "There was no justification whatsoever for these eleven men leaving the sanatorium as they did. As a matter of fact, I hardly think that these complaints were the true cause of the eleven men leaving—they were merely excuses." Misconceptions were bound to arise when patients of this type were led to expect "a first-class hotel" in place of the necessarily strict regime of a sanatorium. Unfortunately, it is not the author of the phrase, but the medical staff of the institutions, who have to pay the penalty for misleading descriptions such as this.

### PANEL DOCTORS RESIGN AT NEWCASTLE.

The question which we ask in another Note as to how many panel practitioners will renew their contracts on April 14, the date predicted by Mr. E. B. Turner in a recent interview in our columns as a test of the hold of panel servitude on the profession, has begun already to be answered in Newcastle. There, we are informed, several medical men have signified their intention of withdrawing from the panel as a protest against the conditions of working the Insurance Act. Their main desire appears to be an income limit of £100. It will be very interesting to compare the demands which are certain to be made in all parts of the country in the course of the next few days, for it must be remembered that these demands have only to be made concurrently and on a reasonably similar scale to form a body of protest which can hardly be ignored. Those who, like Mr. Turner, are fighting hard against the present onus of the panel system, and have done so much to "collect the voices" of insured persons in demand for a fulfilment of the promise of free choice of doctor, have been waiting for the opportunity provided by the expiration of provisional agreements on April 14. The protests which are being made have this additional force behind them that they are the outcome of three months' experience of the panel system, and if the aggrieved insured persons unite with the overworked panel practitioners there is yet a chance of removing the disastrous yoke imposed on both patients and doctors by the panel system

### THE INSURANCE ACT AND SECRET REMEDIES.

The heyday of the proprietary medicine seems a little clouded, and this apart from the popular sales of "Secret Remedies." Of course, the Insurance Act is the cause of the decline. The time was when people preferred to use proprietary medicines instead of incurring the perhaps unknown expense of a doctor's bill, and to have their consultations wrapped round the bottle. Now that medical attendance is supposed to be abundant for those whose compulsory contributions have been already paid, the panel practitioner is receiving a little of the attention which the secret remedy has hitherto received. Conversely, this has led to a revival of dispensing, but it is doubtful whether, under panel servitude, the insured patient gains as much added benefit from visiting his doctor as he ought to do. The reason why is apparent from the following.

### FURTHER FRUITS OF PANEL SERVITUDE.

To be refused by five doctors is an experience which the panel system has inflicted on a porter's wife in the North. A woman, aged thirty-seven, who was expecting her confinement, has, as is not unusual among her class, neglected to engage a doctor, and on being taken suddenly ill sent for Mr. Edmund Barker. As she was not on his list he declined to attend, and when called later stated that he was too much fatigued to visit the case. Four other medical men likewise, we understand, refused to go. Mr. Barker, however, eventually visited the woman, and stated at the inquest, for she died, that his partner and himself had seen one hundred and twenty-five cases the day before, and also that he had been called out of bed six times during the night. They were unable to do all the night work which they did previous to the date of the Insurance Act's enforcement. The Deputy Coroner, after a verdict of "Death from natural causes" had been returned, exonerated the doctor from blame. In little over a week's time arrives the date for renewing, or refusing to renew, the panel contracts. What will the bulk of panel practitioners do?

### MR. PIERPONT MORGAN'S DEATH.

One can hardly be a multi-millionaire without being also a founder of institutions, but the recorder of charities on an immense scale has to choose between a list of gifts so lengthy as to defeat its own end, or a survey which gives little idea of the scale on which a millionaire's generosity is practised. In New York, of course, the site and building of a great maternity hospital were the late Mr. Morgan's gift, Harvard Medical School received a munificent sum from him, as did the Loomis Sanatorium. These benefactions alone amounted to several million dollars. In England the name of Morgan is associated intimately with the Royal National Pension Fund for Nurses, of which Mr. J. P. Morgan was a vice-president, and for the members of which the Junius S. Morgan Benevolent Fund has done such splendid work. Perhaps someone will do for him what has been done for the late Passmore Edwards, and compile an instructive catalogue of his benefactions. We do not think, however, that this is necessary in order to bring out the moral of Mr. Morgan's generosity. No one knew better than he that there is only one thing equally as hard as earning money, and that is spending it usefully. In fact, history seems to show that so to spend requires the higher intelligence. It is not merely a matter of necessity, but of common sense, to sink a millionaire's personality in a great spending department. The personality has its expression in the organising of this department. When funds at an individual's command accumulate to the embarrassing point of multi-millions, we must not be too critical provided that this spending power is organised with an intelligent endeavour to cover the main, and especially the unendowed, departments of human activity. Everything must be done on a national scale, and the collection of art treasures, for example, must aim simply at completeness and catholicity. For we can be too rich as well as too poor to indulge in personal caprice.

### COTTAGE HOSPITALS AND PAYING PATIENTS.

The Horsham Cottage Hospital has taken advantage of its annual meeting to pass a rule which will allow not more than two paying patients to be admitted by special arrangement into the institution. In moving the resolution which embodied this change of rule Mr. Chasemore urged that its adoption would not only increase the income, which is much needed, but would also make the hospital popular, and add to the numbers of subscribers. The resolution was unanimously passed. We have often urged the virtues of a classified system of hospital wards, which would include pay wards of various grades, as has been adopted in America. But until such a system is in universal use, the pay ward in a voluntary hospital naturally presents difficulties. For example, in a town or urban neighbourhood the proposal to introduce a pay ward into a voluntary hospital has brought down before now fierce denunciations of unfair competition from the local nursing home, and when the question of what fee should be charged to paying patients is gone into, it is found very difficult to decide. If the hospital desires to make a profit out of a paying patient its fees must be practically as high as those of the nursing home. If, on the other hand, it is tenacious of its charitable basis, it is limited to a fee of not more than £2 2s., which, however useful in catering for a patient who cannot afford the fees of a nursing home, and yet is above free charity, hardly is profitable in a business sense. We shall be curious to learn the success of the new departure at Horsham Cottage Hospital.

### THE HOLLOWNESS OF SANATORIUM BENEFIT.

Another case of cruel delay in the giving of sanatorium benefit comes to our notice, and is a particularly damaging criticism of the Insurance Act's provisions, because the hardship exists in spite of the good will and attempted co-operation of all the authorities concerned. For the second time the West Derby Guardians had to consider last week the case of a skilled, fully insured man, for whom the Lancashire County Council, through its Insurance Committee, were willing to pay 30s. a week. There being no available sanatorium accommodation elsewhere, the County Council clerk, Mr. Harcourt Clare, asked the Guardians to allot a vacant bed in the Heswall Tuberculosis Joint Hospital, which the Guardians were willing to do. It was then found that the County Council had no power to pay for a patient in a Poor-Law institution, and that the Guardians strictly could not take over the case, as it was not destitute. The former objection is the real one, however, for Poor-Law institutions, at any rate infirmaries, do as a matter of fact admit all sorts of patients, septic cases for example, who are not destitute. Where, it may be asked, are the first-class hotels which were mentioned as awaiting the insured tuberculous patient when the date of benefits arrived? Where indeed, but in the hectic imagination of politicians who have seen promises outrun performance in party politics before now often enough to forget that promises are still believed in by voters. Either the Act must create institutions for the accommodation of such cases as this or it must repeal the clause which forbids payment for patients in Poor-Law institutions, and this it can do only by embarking on some such scheme of co-operation as we outline elsewhere. The empty beds exist, but it requires immediate action and intelligent statesmanship to fill them. In the meantime sanatorium benefit in cases like the present is a hollow sham.

### MEDICAL CERTIFICATES IN IRELAND.

At the end of last week the agreement, which we had previously recorded, between the Irish Insurance Commissioners and the Conjoint Committee of the British and Irish Medical Association on the payment for medical certificates required by Approved Societies and Insurance Committees has been rejected by a meeting of the Irish Poor-Law medical officers in Dublin. The meeting repudiated the action of the secretary of the Conjoint Committee in informing the Commissioners that their scheme would be accepted provisionally by the majority of the profession, and its members refused to go on the panel unless a fee of not less than three shillings is offered. This fee was the one which the delegates asked for in June last for rural areas. Under the provisional scheme hereby repudiated by the Poor-Law doctors the capitation rates in the four areas described last week ranged from 9d. to 2s. for medical certificates.

### IRISH GUARDIANS AND MEDICAL BENEFIT.

Further objections to the present dispensary system in Ireland were heard last week at the resumed sitting of the Treasury Committee in Dublin which is inquiring into the desirability of extending medical benefit to Ireland. Mr. Denis Lynch, chairman of the North Dublin Board of Guardians, and Mr. William Crimmins, a member of the South Dublin Board of Guardians, both favoured the provision of medical benefit if the dependants of the insured were included. The number of dispensary patients would be very much reduced by it, and the dispensary scheme was objected to by the poor, who would like to get rid of the Poor-Law system. Mr. J. Cotter, clerk of the Cork Union, was also in favour of the break-up of the dispensary system, which, as we have previously pointed out, for stated reasons is unsatisfactory for both patient and doctor—one is badly treated, the other badly paid. The opinions of such officials as the above represent a very large mass of opinion.

### THE ROGERS PRIZE FOR 1914.

Under the will of the late Nathaniel Rogers, the University of London offers from time to time a prize of £100, open to all members of the medical profession in the United Kingdom, for an essay or dissertation on some subject of medical or surgical interest chosen by the University. The prize will be competed for in 1914, and the subject named by the University is "The Nature of Pyrexia and its Relation to Micro-Organisms." The problem thus suggested is not very likely to receive any exact or complete solution from the competitors. Indeed, it will probably be many years before a scientifically proved theory of pyrexia, and of the way in which organisms provoke it, is accepted as satisfactory. But it will be very interesting when the time comes to see what the essayists have made of the matter; and it may be even more interesting in twenty years' time to look back and see how far their suggestions stand the test of time and research. There is, if we may say so, a thoroughly sporting element about the chosen subject; and this is not a drawback, because it affords also an excellent chance of reviewing recent progress and of intelligently anticipating future work.

### TAXATION ON GIFTS TO CHARITIES.

Mr. William Jones, solicitor to King Edward VII.'s Hospital at Cardiff, has made out a strong case against the taxation of charitable gifts left to hospitals and other public institutions ministering to the sick. He declares that public opinion is gaining ground and that the time has arrived when the 10 per cent. duty imposed upon all charitable bequests should be removed in the interests of the suffering poor and national health. Mr. Jones maintains that many members of Parliament known to him have promised to interest themselves in this important question, seeing that the needs of the hospitals all over the country were never greater than now. The present legacy duty was imposed in 1853 with the object of taxing *post-mortem* gifts "to strangers in blood to the testators," and, although charitable institutions were then included, the law should be speedily amended. He points out that in the case of a gift of £300 made in the donor's lifetime in April last to the Cardiff Hospital, the giver having died within a year, the Government are enforcing their claim for legacy duty. He expresses the hope that there is nothing definite or final about the Chancellor's announcement to the British Hospitals Association that he could not submit to Parliament legislation exempting hospitals from death duties. Certainly the ground for this decision given by Mr. Lloyd George—*i.e.* the loss of revenue—will not bear close examination. The truth is the voluntary hospitals have the remedy in their own hands. Directly through the members of Parliament for the district each hospital serves and indirectly through the British Hospitals Association continuous pressure should be put upon Parliament and the Government until this unjust taxation is removed from the voluntary hospitals. Energetic action is the one thing needful. Will it be forthcoming?

### INSURANCE ACT'S EFFECT ON HOSPITAL INCOME

Hospitals on the whole have been prompt to issue statistics informing their subscribers how much their income and how much their expenses were likely to be affected by the Insurance Act. The Kensington and Fulham General Hospital, however, appears to have gone one better. Advantage of the annual meeting was taken to publish the variation in income which coincided with the date on which the Insurance Act came into force. From January 1, 1912, to June 30 the income was given as £1,113, while from July 1 to December 31 the income fell to £788. And this though the autumn quarter is generally regarded by hospital secretaries as the best. Whether the idea of juxtaposing these two sets of figures sprang from the fertile brain of Mr. Louis McCausland, the hospital secretary, we do not know, but the moral of the figures can hardly be lost on the supporters of an institution which has set itself such a big task of reconstruction as this. We hope that moral will not be wasted.

### THE CRY FOR CENTRALISING RESEARCH.

The proposal in the Departmental Report on Tuberculosis which appears to have excited most discussion is that concerning a Central Bureau. Ardent reformers, expanding this suggestion, have hastened to put forward the advantages of a Central Research Institute where every facility would be offered, and urged that this might act, in addition, as the Central Bureau, which was all that the report actually proposed. In view of the existing cancer and tuberculosis hospitals—all of which, we may say, have research departments, trained staffs, and patients close at hand—it is hard to see how a Central Research Institute could improve matters. While there is a good deal to be said for a Central Bureau, in which the results of investigations and all records of experiment could be filed, we are sure that no good could be gained by attempting to replace the existing research departments of our special hospitals by an institution which would labour under the supreme disadvantage of being removed from the patients whose specimens it would have to investigate.

### CHIEF CLERK OF BETHLEM HOSPITAL RESIGNS.

Many sides of institutional life in London are feeling the resignation of Mr. William Hipwell, who has been for eighteen years chief clerk of the Bethlem Royal Hospital. His birth and life are closely connected with London, for he was born here, and educated at University and King's Colleges. The many interests of his life are shown by his connection with various societies and institutions. He is a governor of the City of London Lying-in Hospital, vice-president of the Asylum Workers Association, and honorary local secretary of the After-Care Association for Poor People Discharged Recovered from Mental Hospitals, and a member of the Hospital Officers Association. Mr. Hipwell has also engaged in municipal and Parliamentary electioneering, and is a Fellow of the Zoological Society. Volunteering has been another of his active enthusiasms, which we hope, since his resignation has occurred, will find an ample leisure for their free exercise, now that the strain of institutional responsibility has been removed.

### REFORMS AT CHELTENHAM HOSPITAL.

The Report of the Special Committee which has been investigating the management and financial position of the General Hospital, Cheltenham, has been adopted, save for one particular, by the majority of the subscribers at the recent annual meeting. It will be remembered that what struck competent visitors most unfavourably for several years past has been the quarters set apart for the nurses. A drastic change at last has been decided on to put this right. The private nursing staff has been abolished, for there are, it appears, no funds in hand to rebuild the present nurses' quarters, and therefore it has been decided to appropriate for them the quarters of the private nursing staff. For financial reasons the branch dispensary is also to be closed, though last year some five thousand cases received treatment. It must be remembered that of late years special efforts have succeeded in reducing the annual excess of expenditure only by one-half, from £4,000 to £2,000, and this retrenchment of activity, as the closing of the branch dispensary may be termed, follows the closing of forty beds. Dr. Cardew, the chairman of the Special Committee, in proposing the abolition of the private nursing staff, is reported to have said that, even were that done, it would not be possible to give every nurse a bedroom to herself. Another change, of a personal nature, is the retirement of Colonel Croker-King from the presidency, which he has held for twenty years. Even before this he was an active worker for the institution, having been for six years honorary secretary and treasurer. He leaves the institution at the parting of new ways, which we must all hope will lead to better administration and increase the confidence of the subscribers. Such an increased confidence is proved in the long run to be the only way of enlarging their numbers.

### BIRMINGHAM'S NEW CHIEF MEDICAL OFFICER.

The Birmingham Board of Guardians, under the chairmanship of Mr. Frank Jackes, have appointed Mr. F. W. Ellis, F.R.C.S., resident medical officer at Selly Oak Infirmary, chief medical officer. There were two other candidates, one a London and the other a Birmingham surgeon. The appointment of Mr. Ellis was moved by the Chairman of the Infirmaries Committee, Mr. Darby, and seconded by Mr. Mackenzie. Mr. Ellis' appointment is, of course, a recognition of the extreme value and importance of Poor-Law infirmary work in candidates for posts in other branches of the Public Health Service.

### MEDICAL OFFICER ASKED TO RESIGN.

Dr. W. Arnold Evans, medical officer of health for Bradford, who has been asked to resign by the Bradford Health Committee, was born in 1862, and has held his present appointment for twenty-two years. He was educated at Owens College, and has held the posts of physician and pathologist at Manchester Royal Infirmary. The Chairman of the Health Committee which decided to request Dr. Evans' resignation is Mr. E. J. Smith, and the decision of the committee was arrived at only after a protracted discussion lasting several hours. The resolution embodying the decision was eventually carried by six votes to four. Mr. Evans, who is a B.Sc., qualified M.R.C.S. in 1883, and is a Fellow of the Sanitary Institute. It is unfortunate that a grave decision of this kind has passed without all the facts of the case, which the voting shows to have been open to two interpretations, being laid before the bar of public opinion quite fully. Frankness in these matters alone can guard against public misconception and private bitterness.

### THE NO-COLLAR CRUSADE.

A year or two ago we gave prominence in these columns to the views of Mr. W. G. Walford upon the injudiciousness of tight neck-wear. To collars of every kind he entertains a rooted antipathy, and attributes a long train of disorders in remote parts of the body to the direct or indirect results of constriction about the neck. In a recent communication Mr. Walford sends us details of a lady who had suffered for ten or twelve years from menorrhagia, which was getting worse. She had had one abdominal and several vaginal operations for this trouble, but could not get relief. At last she applied to Mr. Walford, and on following his directions as to neck coverings she very greatly improved within two months. The directions are that every scrap of clothing above the collar-bone should be so loose that the whole hand can be passed down inside it. By this simple device its inventor believes that an enormous amount of ill-health can be prevented. Obviously, it entails a disregard of fashion and convention quite beyond the ordinary, which probably explains the fact that no very great number of converts have as yet been made.

### THE CHURCHES AND HOSPITAL HOUSEKEEPING.

It has been mentioned on more than one occasion in this Journal that, as a substitute for the annual flower service, in isolated cases "Marmalade," "Egg," or "Toy" services have been held with good results, at which articles of practical use have been substituted for the flower with its somewhat precarious future existence. Were the suggestion more universally adopted by clergymen or by institutional workers, something would be done to reduce the hospital housekeeping bill. Attached is a photograph of the result of such a service held in a poor district in London-over-the-Border. This photo shows the gifts at a "Marmalade and Toy" service at St. Gabriel's Church, Walthamstow.

### FROM SENIOR SURGEON TO DEPUTY CHAIRMAN.

The resignation of Mr. C. J. Bond, F.R.C.S., of the post of senior surgeon to the Leicester Royal Infirmary has been received with the keenest regret. No other proof of esteem was needed than the election of Mr. Bond to the deputy chairmanship of the institution in the place of Sir Arthur Grey Hazlerigg, who is not seeking re-election to a position which he has held for several years. Mr. Bond has had long and useful association with the institution. Appointed house surgeon in 1882 his active connection has remained unbroken until the present time. In 1886 he was appointed assistant surgeon, and a few months later honorary surgeon, in which capacity the board acknowledge with gratitude his work—in conjunction with the late Sir Charles Marriott—in the introduction of modern aseptic methods into the infirmary. "By his address on surgery at the British Medical Association annual meeting in Leicester in 1905, and by his masterly opening of the discussion on septic peritonitis in the Surgical Section of the Association's Toronto Meeting in 1906," the work performed in Leicester became widely recognised. His contribution to surgical literature and his writings on heredity are well known. To mark their appreciation, Mr. Bond's name will be submitted to the next quarterly meeting of the governors for election as an honorary consulting surgeon and a vice-president of the institution.

### SAVERNAKE HOSPITAL AND THE MAURICE MEMORIAL.

The announcement made by Dr. W. B. Maurice at the recent annual meeting of Savernake Hospital that Mr. Ernest Wills, of Ramsbury Manor, had promised to pay off the deficit of some £300 should put new heart into the subscribers. We have previously alluded to the fine work of this institution, which has steadily grown from small beginnings, and is typical of the immense value which a cottage hospital can be when guided by personal enthusiasm. The late Dr. J. B. Maurice was pre-eminently an example of this and of the general practitioner who gave to the sick in his locality surgical treatment that would have been thought impossible in a cottage hospital years ago. The Memorial Fund which has been raised to him now reaches some £370, and we hope that the hospital's supporters will make it their business that at least £500 is raised before the second anniversary of Dr. Maurice's death comes round.

### THE COMMUNION CUP.

We have dealt before now with the alleged infective dangers of the Communion cup, but these alleged dangers are a hardy annual, and so, as the common sense of the matter can be briefly stated, we may summarise them once more. That the transmission of infection can be conveyed through the medium of a common drinking vessel is undoubted, though why it is always the chalice rather than the drinking-fountain cup which is criticised we do not know. It is again true that individual vessels for each communicant would do away with the risk, and as far as we know there is nothing theologically which can be urged against their use, except the modification of customary practice. Much capital is made by advocates of the individual chalice of the fact that the Apostles in Leonardo's "Last Supper" at Milan are depicted as provided with one cup apiece, and it is probable that this practice obtained in early Christian times. But it is with the medical aspect of the question which we are here concerned, and the truth is that no statistics are available from which to learn how far the theoretical danger is a practical one. As, however, there seems little, if any, theological objection to the individual chalice, and an undoubted theoretical objection to the single one, we can disregard the alarmists and hope that a common-sense precaution will commend itself to clergy and communicants.

### THE PRICE OF EXEMPTION.

The Lambeth Board of Guardians have not adopted the suggestion of the Local Government Board, referred to in The Hospital on March 15, that vaccination officers should receive fixed salaries. The infirmary committee reported that the proposal has the disadvantage of not inducing vaccination officers to secure the vaccination of children wherever possible. They consider the present method of "payment by result" to be the best; but, as the number of persons obtaining exemption certificates is rapidly increasing, and likely still further to increase, they propose that the Local Government Board be urged to sanction the payment to vaccination officers of a fee for every case in which they received an exemption certificate. It was suggested that the fee should be equal to three-quarters of the sum payable to the officers in cases of successful vaccination. The Board agreed to the proposal.

### PROPOSED REGISTRATION OF DISPENSERS.

A Bill dealing with the question of the registration of dispensers is in course of preparation. It will be recalled that in the House of Commons during the Committee stage of the National Insurance Bill Mr. W. S. Glyn-Jones, M.P., the Pharmaceutical Society's Parliamentary Secretary, said it was the intention to promote legislation dealing with the qualification of dispensers, and it is in pursuance of this pledge that the Bill is being prepared. The Parliamentary and General Purposes Committee of the Council of the Pharmaceutical Society has already prepared a draft scheme, and is consulting the General Medical Council, the Apothecaries' Society, and the Home Office in connection therewith. It is proposed to set up a register of persons entitled to assist pharmacists in dispensing medicines, and when the proper time arrives the whole question of the position of dispensers under the Insurance Act will be considered.

### BACTERIOLOGY FOR HOSPITAL PHARMACISTS.

Dr. R. T. Hewlett, Professor of Bacteriology at King's College, has concluded a series of lectures on "Micro-biology and Pathological Chemistry and Microscopy in Relation to the Pharmacist." As these lectures were arranged for by the Pharmaceutical Society's Council it seems not improbable that at a future date the subject may be introduced into the pharmaceutical curriculum. A knowledge of bacteriology might be useful to hospital pharmacists, who, if adequately trained, might be able to assist hospital practitioners by making investigations of morbid material, and if the Pharmaceutical Society establishes the course of instruction outlined by Professor Hewlett it would no doubt be well attended.

### A NEW HOSPITAL AND LOCAL ENTHUSIASM.

When the new Nelson Hospital for Wimbledon, which succeeds the South Wimbledon, Merton and District Cottage Hospital, was opened, fears were expressed by some members of the committee that funds to defray the increased expenses would not be forthcoming. The grounds for this were mainly that the Cottage Hospital had fourteen beds, while the present hospital was intended for twenty-four. The patients were transferred to the new building on Easter Tuesday 1912, but within three weeks two extra beds had to be provided. As was estimated the annual expenditure has been about double that of recent years, but thanks very largely to a grant of £250 from King Edward's Fund for maintenance, to a substantial increase in patients' payments, and to local entertainments, organised on behalf of the hospital, these fears have proved ungrounded. The annual subscriptions have much improved, and we are glad to know that a very great deal of interest in the hospital exists in the neighbourhood. The above fears are typical of those which attend the start of every new or enlarged institution. Where there has been a real need and good management they vanish cheerfully as in this case.

### A SOLID MEDICAL AUTHOR.

The death of Dr. Alfred Lewis Galabin removes not merely a brilliant scholar of a type not so common as it once was, nor merely a distinguished obstetrician, but the author of a famous treatise. Galabin's "Manual of Midwifery" has the solid virtues of a scholarly mind, and though the years inevitably modify cherished medical, as other, dogmas, and the axioms of one generation are questioned by the next, it will long remain the model and type to which later manuals must conform however much they may prune or expand the matters which it embodies. When we have still to lament the lack of literary style in many text-books, it is well to remember that the author of the "Manual of Midwifery" was, in the golden manner of nineteenth-century Cambridge scholars, a first-class classic and a Wrangler, as well as a doctor of medicine. The year 1866 was the date of his scholastic triumphs. Later, of course, he became a Fellow of the Royal College of Physicians and has been president of the Hunterian and Obstetrical Societies. Dr. Galabin holds that place, which is especially dear to the true scientist, of an author on whom the student can be nourished, whoever else he may turn to in a maturer stage.

### LONDON'S NEW MENTAL HOSPITAL.

The Mental Hospitals Committee of the London County Council report that tenders were recently invited from selected firms for the erection on the Horton Estate at Epsom of the eleventh Mental Hospital for the County of London. Eleven tenders were received, and the Committee have accepted the lowest tender submitted—that of Messrs. Leslie and Co., Ltd.—at the total of £335,577, and the solicitor has been instructed to complete the contract, subject to the approval of the Home Secretary.

### UNLICENSED HOUSES AND MENTAL PATIENTS.

The important allegation of receiving mental patients for payment in an unlicensed house was one among several grave charges made against a medical man this week. It is hardly necessary to remind our readers that the Commissioners in Lunacy, who issued the summonses in the present case through the Public Prosecutor, have no right to visit uncertified patients, and that the enforcement of the licensing provisions is of very great importance.

## Figures and Tables

**Figure f1:**